# A simplified method for analysis of polyunsaturated fatty acids

**DOI:** 10.1186/1471-2091-6-5

**Published:** 2005-03-24

**Authors:** Jing X Kang, Jingdong Wang

**Affiliations:** 1 Department of Medicine, Massachusetts General Hospital and Harvard Medical School, Boston, MA 02114, USA

## Abstract

**Background:**

Analysis of fatty acid composition of biological materials is a common task in lipid research. Conventionally, preparation of samples for fatty acid analysis by gas chromatography involves two separate procedures: lipid extraction and methylation. This conventional method is complicated, tedious and time consuming. Development of a rapid and simple method for lipid analysis is warranted.

**Results:**

We simplified the conventional method by combining the extraction and methylation into a single step (omitting the procedure of prior extraction). Various biological samples including cultured cells, animal tissues and human specimens have been tested using the new method. Statistical analysis indicates that the recovery of long chain fatty acids from tissue samples by the simplified method is significantly higher than that by the traditional method, but there is no difference in relative fatty acid composition between the two methods. This simplified method can significantly save time and materials, and reduce the potentials of sample loss and contamination.

**Conclusion:**

The lipid extraction procedure prior to methylation employed conventionally in lipid analysis can be omitted without affecting the recovery of long chain (≥ 18 C) fatty acids and their composition. The simplified method is rapid, easy-to-use, suitable for analysis of total long chain polyunsaturated fatty acid contents (e.g. n-6 and n-3 fatty acids) in various biological samples, especially when the number of samples to be analyzed is large and/or the specimen size is small.

## Background

Fatty acid composition of cell membrane is an important determinant of cell function [1]. Manipulation of cellular fatty acid composition has been a widely used approach to modulating the biological responsiveness of different cell types. Recently, fatty acid profile, particularly the ratio of omega-6 (n-6) to omega-3 (n-3) polyunsaturated fatty acids, of cells or tissues has become a biomarker for monitoring the outcome of dietary interventions (i.e., fatty acid supplementation) and for identifying the risk factors for lipid related diseases (e.g. cardiovascular disease) [2]. Measurement of the n-6/n-3 fatty acid ratio can be also used to identify animal phenotypes, such as the fat-1 transgenic mice that we created recently [3]. Thus, analysis of fatty acid composition is a commonly used technique in lipid research.

Analysis of fatty acid composition is usually carried out by gas chromatography (GC). Conventionally, preparation of samples for GC involves two separate procedures: extraction and methylation. Lipids are usually extracted from cells or tissue homogenates by using organic solvents such as chroloform/methanol [4]. This procedure is time and material consuming, potentially causes sample loss and contamination, and generates organic wastes. These problems become more apparent when the number of samples to be extracted and analyzed is large and/or the specimen size is small.

This study validated a method that combines the extraction and methylation into a single step. Our results showed that this simplified method without the need of prior extraction yielded desirable outcome.

## Results and discussion

**Figure 1:**
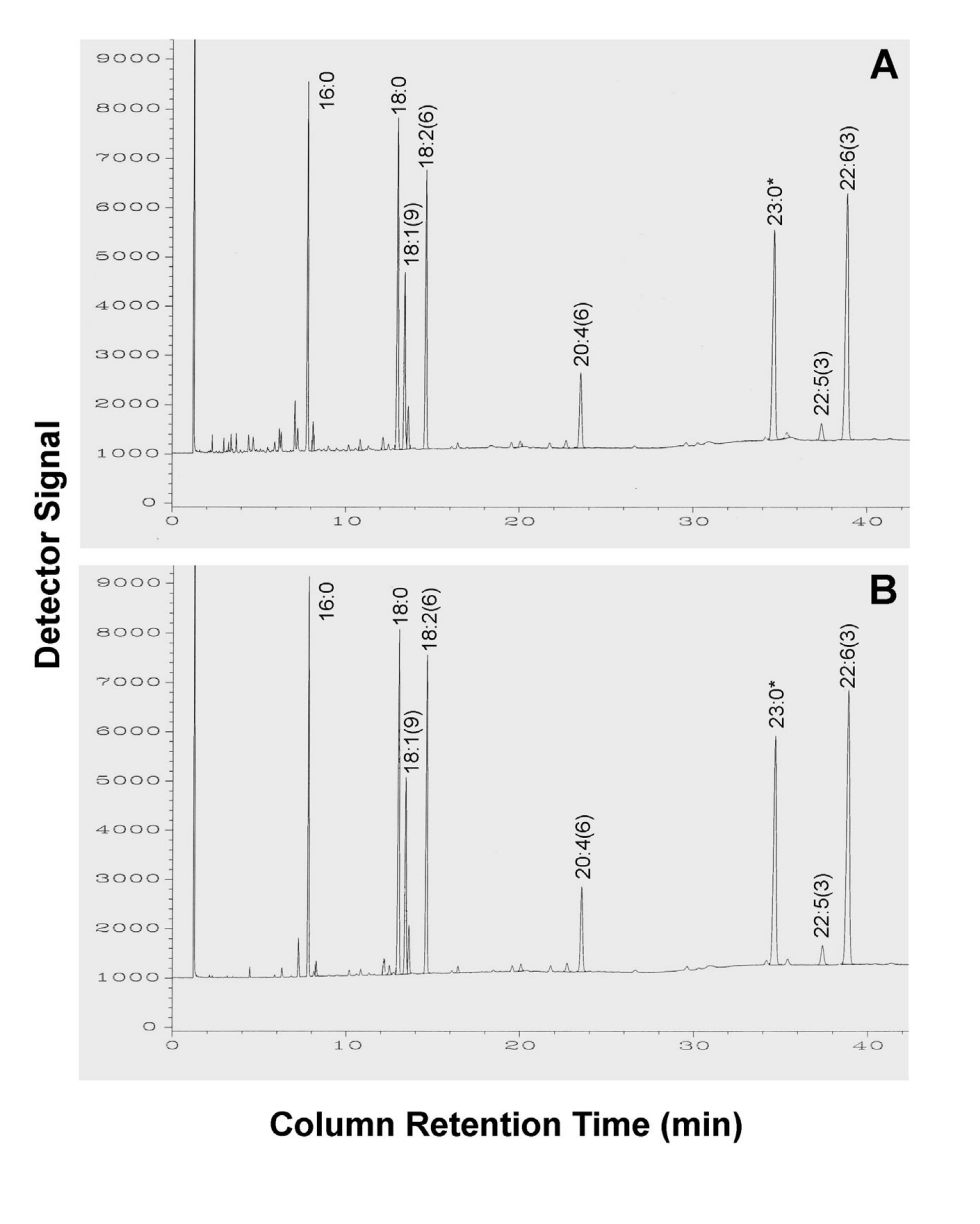
Comparison of the results (gas chromatographs) of lipid analysis by the conventional and simplified methods. Mouse heart tissue was homogenized by grinding it up in liquid nitrogen and same amount (2 mg/sample) of the homogenate was used for analysis by each method under the same chromatographic conditions, as described in the Methods. Panels A: The GC result obtained using the conventional method. Panel B: The result obtained using the simplified method. Note, the fatty acid 23:0 (1 μg) was added to the sample before extraction as an internal standard.

The samples tested, including cultured cells, tissue homogenates, and red blood cells, were divided into two aliquots. One was analyzed by the conventional method and the other analyzed by the simplified method, under the same chromatographic conditions. The results obtained using the conventional and simplified methods were compared. Figure 1 shows a representative set of the fatty acid profiles of mouse heart tissue derived from the two methods. Generally, the results obtained using the simplified method without prior extraction are as good as or even better than those obtained using the standard method, particularly when the sample size is small. As shown in Table 1, the recovery of long chain fatty acids from tissue samples by the simplified method is significantly higher than that by the traditional method, but there is no difference in relative fatty acid composition between the two methods. However, it was noted that some short and medium chain fatty acids (<16C) could be lost, but none of those fatty acids with a chain length of 16 or more carbons was affected (Fig. 1). Similar outcomes were found in the tests using cultured cells, red blood cells and mouse-tails. These results indicate that the extraction of lipid prior to methylation is not necessary for the analysis of total long chain fatty acid composition by GC. We also found that the water in liquid samples, if its content is not more than 5% of the volume of BF3 solution added, did not affect significantly the analytic outcome. Therefore, the simplified method is suitable for both dried and liquid samples. However, the result obtained using the simplified method is limited to fatty acid profile of total lipids in the analyzed specimens. For studies requiring fatty acid composition of individual lipid class (e.g. phospholipid or triglyceride), prior extraction and separation of lipids are still necessary for their analysis.

**Table 1:** Quantitative results of lipid analysis by the conventional and simplified methods. Sample preparation and lipid analysis were performed as described in Fig.1. The quantity (absolute amount) of each fatty acid is represented by its peak area. (The initial weight of tissue samples for the two methods was the same.) The area percent of each fatty acid was calculated by dividing its peak area by the total peak area of the 8 fatty acids identified (excluding 23:0). Values are means ± SD of five (n = 5) measurements. Values for each fatty acid with the same letter do not differ significantly (p < 0.05) between conventional and simplified methods.

**Fatty Acids Identified**	**Quantity **(Area of Peak: × 1,000 counts)		**Composition **(% of total LCFA identified)	
	
	**Conventional Method**	**Simplified Method**	**Conventional Method**	**Simplified Method**
**16:0**	26.3 ± 0.8^a^	30.4 ± 0.6^b^	14.6 ± 0.1^a^	14.5 ± 0.6^a^
**18:0**	34.5 ± 1.2^a^	37.5 ± 0.4^b^	18.9 ± 0.2^a^	18.5 ± 0.3^a^
**18:1(9)**	18.1 ± 0.6^a^	21.6 ± 0.3^b^	10.1 ± 0.1^a^	10.3 ± 0.1^a^
**18:2(6)**	29.3 ± 0.9^a^	35.3 ± 0.5^b^	16.3 ± 0.2^a^	16.8 ± 0.3^a^
**20:4(6)**	12.7 ± 0.5^a^	15.0 ± 0.2^b^	7.0 ± 0.2^a^	7.1 ± 0.1^a^
**22:5(3)**	4.1 ± 0.2^a^	5.0 ± 0.3^b^	2.1 ± 0.1^a^	2.3 ± 0.2^a^
**22:6(3)**	55.1 ± 1.6^a^	64.4 ± 1.3^b^	30.6 ± 0.2^a^	30.7 ± 0.3^a^
**23:0 (std)**	42.9 ± 1.6^a^	47.7 ± 1.0^b^		

## Conclusion

The present study has demonstrated that the lipid extraction procedure prior to methylation employed conventionally in lipid analysis of long chain (≥ 18 C) polyunsaturated fatty acids in biological samples can be omitted, without affecting the recovery of long chain fatty acids and their composition. This simplified method is suitable for analysis of long chain fatty acid composition in a variety of biological specimens, but not appropriate for quantification of medium and short chain (<16C) fatty acids. Because the modified method is relatively simple and sensitive, it has a number of advantages including saving time and easy-to-use, reducing the potentials of sample loss and contamination, and requiring only small quantities of specimens and solvents. These advantages become more obvious in the case where the number of samples to be analyzed is large and/or the specimen size is small. For example, use of the simplified method would make the task of lipid analysis much easier in the large clinical trials that need to monitor polyunsaturated fatty acid composition (especially n-3 fatty acid contents) of red blood cells or other specimens from a large number of subjects. This new method is particularly useful for phenotype analysis of the transgenic animals that exhibit a unique fatty acid profile, such as the fat-1 transgenic mice that we generated recently [3].

## Methods

### Conventional method

Cell or tissue lipids were extracted by the procedures similar to the Folch method [4]. Chloroform/methanol (2:1, v/v) containing 0.005% butylated hydroxytoluene (as antioxidant) was added (usually 5 ml solvent added to 50–100 μl sample) and mixed vigorously for 1 min then left at 4°C overnight. One ml of 0.9% NaCl was added and mixed again. The chloroform phase containing lipids was collected. The remains were extracted with another 2 ml chloroform. The chloroform was pooled and dried under nitrogen and subjected to methylation. To monitor the recovery rate, the fatty acid C23:0 was added to the samples (usually 1 μg added to 2 mg tissue sample) as an internal standard.

Fatty acid methyl esters were prepared by methods similar to those described previously [5,6] using BF_3_/methanol reagent (14% Boron Trifluoride). Lipid sample was mixed with 1 ml hexane in 16 ml glass tubes with Teflon-lined caps. BF_3_/MeOH reagent (1 ml) was added and the mixture was heated at 90–110°C in a metal block or a sand bath for 1 hour, cooled to room temperature and methyl esters extracted in the hexane phase after addition of 1 ml H_2_O. Samples were allowed to stand for 20–30 min, and then the upper hexane layer was removed and concentrated under nitrogen.

Fatty acid methyl esters were analyzed by gas chromatography using a fully automated HP5890 system equipped with a flame-ionization detector, as described previously [7] The chromatography utilized an Omegawax 250 capillary column (30 m × 0.25 mm I.D.). Peaks were identified by comparison with fatty acid standards (Nu-chek-Prep, Elysian, MN), and area and its percentage for each resolved peak were analyzed using a Perkin-Elmer M1 integrator.

### Simplified method

An aliquot of cell pellet or tissue homogenate (<50 μl) in a glass methylation tube was mixed with 1 ml of hexane and 1 ml of 14% BF_3_/MeOH reagent. After blanketed with nitrogen, the mixture was heated at 100°C for 1 hour, cooled to room temperature and methyl esters extracted in the hexane phase following addition of 1 ml H_2_O. The samples were centrifuged for 1 minute, and then the upper hexane layer was removed and concentrated under nitrogen. Fatty acid methyl esters were analyzed by gas chromatography as described above.

### Statistical analysis

The results from the two methods were compared by using unpaired t-test, and *P *values of <0.05 were considered significant. Results are means ± SD.

## Authors' contributions

**JXK **conceived of the study, participated in design and conduct of the experiments, and prepared the manuscript.

**JW **carried out sample preparation, GC analysis and data collection.
